# Cardiac involvement and clinical follow up of patients with hereditary transthyretin related amyloidosis associated with Glu89Gln mutation

**DOI:** 10.1186/1750-1172-10-S1-P54

**Published:** 2015-11-02

**Authors:** Mariana Gospodinova, Stayko Sarafov, Velina Guergueltcheva, Andrey Kirov, Teodora Chamova, Albena Todorova, Ivailo Tournev, Stefan Denchev

**Affiliations:** 1 Medical Institute of Ministry of Interior, Clinic of Cardiology, 1606, Sofia, Bulgaria; 2 University Hospital Alexandrovska, Clinic of Neurology, 1431, Sofia, Bulgaria; 3 University Hospital Sofiamed, Neurology Department, 1528, Sofia, Bulgaria; 4 Genetic lab, Genika, 1113, Sofia, Bulgaria

## Background

Cardiac involvement is common in hereditary transthyretin – related amyloidosis (ATTR), but there is a significant phenotypic heterogeneity depending on the mutation.

## Patients and methods

We evaluated forty consecutive ATTR patients with Glu89Gln mutation, focusing on cardiac involvement - 18 male, 22 female at a mean age of 57.6±6,7 years. A clinical examination, 12–channel ECG, conventional 2D, Doppler and tissue Doppler echocardiography were performed. The patients were followed for 36 months in the range from 2 to 78 months.

## Results

Median age of symptoms development was 52, 3±6, 4 years. Cardiac onset was found in 5 (12,5%) patients. Cardiomyopathy and peripheral polyneuropathy were evident at diagnosis in all patients. Echocardiography revealed a significant increase in wall thickness of both left and right ventricles (septum – 18,6±3,4 mm; posterior wall – 17,5±2,5 mm; RV free wall – 8,4±2,0 mm). Varying degrees of LV diastolic dysfunction were found – Grade 1 in 11 (27,5%) patients, Grade 2 in 12 (30%) and Grade 3 in 17 (42,5%) patients. A reduced LV ejection fraction was found in 9 (22,5%) patients. A common finding were significantly reduced mitral annulаr systolic velocities (s´septum-5,4±2,0 cm/s, s lat.-5,7±1,9 cm/s), registered in all the evaluated patients, pointing to an impaired LV longitudinal systolic function. The systolic myocardial velocities of the tricuspid annulus and TAPSE values were reduced respectively 6,9±2,1cm/s and 12,8±3 mm in 14 of the patients (35%). Pericardial effusion was found in 13 (32,5%) patients.

Pathological ECG was present in 35(87,5%) of the evaluated patients. Atrial fibrillation was registered in 4 (10%) patients, A-V block first degree in 8 (20%), low voltage in 15 (37,5%), left bundle branch block in 3 (7,5%), left anterior fascicular block in 9 (22,5%), pathological Q wave in 14 (35%), right bundle branch block in 2 (5%), and pace-maker rhythm in 2 (5%). Rhythm and conduction disturbances on ECG were found in 24 patients (60%).

The following events occurred during the follow-up period: two deaths (5,4%) (one patient due to ischemic stroke; and another due to heart failure). Two other patients suffered from ischemic strokes. 24-hour Holter ECG revealed short periods of atrial fibrillation and an oral anticoagulant was initiated. A sinus pause > 3 s was observed in one of the patients and a permanent pace-maker was implanted. Four new cases (10%) with symptomatic heart failure, requiring diuretic treatment were observed. In 15 patients a worsening of the symptoms from the peripheral neuropathy were found.

## Conclusion

Our study confirms that ATTR associated with the Glu89Gln mutation has a mixed phenotype – neurological and cardiac and an unfavorable prognosis. Our findings imply that patients and carriers of Glu89Gln require close multidisciplinary (both cardiological and neurological) follow-up in order to initiate treatment in time.

